# Introducing artificial intelligence and sperm epigenetics in the fertility clinic: a novel foundation for diagnostics and prediction modelling

**DOI:** 10.3389/frph.2025.1506312

**Published:** 2025-02-27

**Authors:** Adelheid Soubry

**Affiliations:** Epigenetic Epidemiology Lab, Department of Human Genetics, Faculty of Medicine, KU Leuven—University of Leuven, Leuven, Belgium

**Keywords:** ART, sperm, pregnancy success, epigenetic biomarkers, machine learning, biostatistics

## Abstract

Worldwide, infertility is a rising problem. A couple's lifestyle, age and environmental exposures can interfere with reproductive health. The scientific field tries to understand the various processes how male and female factors may affect fertility, but translation to the clinic is limited. I here emphasize potential reasons for failure in optimal treatment planning and especially why current prediction modelling falls short. First, Assisted Reproductive Technology (ART) has become a mainstream solution for couples experiencing infertility, while potential causes of infertility remain unexplored or undetermined. For instance, the role of men is generally left out of preconceptional testing and care. Second, regularly used statistical or computational methods to estimate pregnancy outcomes miss important biological and environmental factors, including features from the male side (e.g., age, smoking, obesity status, alcohol use and occupation), as well as genetic and epigenetic characteristics. I suggest using an integrated approach of biostatistics and machine learning methods to improve diagnostics and prediction modelling in the fertility clinic. The novelty of this concept includes the use of empirically collected information on the sperm epigenome combined with readily available data from medical records from both partners and lifestyle factors. As the reproductive field needs well-designed models at different levels, derivatives are needed. The objectives of patients, clinicians, and embryologists differ slightly, and mathematical models need to be adapted accordingly. A multidisciplinary approach where patients are seen by both, clinicians and biomedically skilled counsellors, could help provide evidence-based assistance to improve pregnancy success. Next, when it concerns factors that may change the ability to produce optimal embryos in ART, the embryologist would benefit from a personalized prediction model, including medical history of the patient as well as genetic and epigenetic data from easily accessible germ cells, such as sperm.

## Introduction

According to an estimate by the World Health Organisation (WHO), fifteen percent of reproductive-aged couples worldwide are affected by infertility (1). The contribution of the male partner is not negligible. Depending on the geographical region, estimated infertility due to men ranges from 20% (in Sub-Saharan Africa) to 70% (in the Middle East). Rates of male factor involvement in Western regions lays in between these two extremes, in Europe this rate is 50% (2). Moreover, after excluding hormonal disorders, anatomical or functional problems (such as varicocele), sexual problems, chromosomal abnormalities or genetic diseases, it has been reported that 70% of male infertility remains unexplained (3). Assisted Reproductive Technology (ART) is one way to overcome this issue. ART is frequently used, but it is certainly not the ultimate solution for all. Since its introduction in 1978 (4), more than 10 million people have been born through this technique worldwide (5). But, as the world population is estimated to drop with 50% before the end of this century (6), ART seems to become only a drop in the ocean. At its current (success) rate it will not solve the burden of rapid decrease in world population growth. The reason for this is diverse. First, limited countries offer public funding for fertility treatment. Hence, this treatment is not affordable for all economic classes of the society. Next, even in wealthy countries, not all couples suffering from infertility attend the clinic and drop-out rates after a first consult are 50%. Reasons for withdrawal are emotional distress and a seemingly poor prognosis (7). Considering clinical studies, it takes 3 ART cycles to reach a success rate of 50% (8–11). Not all patients undergo three cycles. Consequently, fertility treatment provides help for far less than 50% of couples suffering from infertility and most of these couples remain childless (10, 12, 13). In other words, ART treatment, fertility health care and current prevention measures (if applied) have reached their limits. Ideally, fertility care should encompass prevention, diagnosis and treatment of infertility. The current work provides a brief overview of the (economic) burden and highlights some missed opportunities to combine scientific knowledge, epidemiology, mathematical modelling methodologies and clinical care. On-going efforts in other clinical areas where Artificial Intelligence (AI)-based tools have been implemented already will be discussed and new insights will be added to improve quality of patient care in centres for reproductive medicine.

## Epidemiology of male infertility and the price of ART: are we overselling ART treatment in its current form?

A recent report estimated that the total costs for one cycle with a fresh embryo leading to a live birth varied between 4,108 and 12,314 euro, depending on the country. New birth means new future taxpayers and hopefully fewer impact of the growing ageing population (14). Although we do not believe that fertility treatment will solve the general decline of the human population, it may have some advantages economically, in most countries. However, from a treatment perspective, a failure that exceeds 50% is tremendous. Next to the psychological distress couples encounter before and during treatment, fertility treatment is a financial ruin, for patient and government. Would the same failure rate still be acceptable in other clinical domains, such as in effectiveness of new vaccines or in development of new therapeutic drugs? Additional financial consequences -not always included in estimates of ART cost- are related to the procedure itself, such as ovarian hyperstimulation syndrome, premature delivery, absence from work, and doctors' visits related to psychological distress, especially if ART fails (15–17). While cost-effectiveness of fertility treatment is not the aim of this work, it would be worth to estimate also beneficial effects of infertility prevention. If governments put more resources on prevention in young men and women, invest in research on better clinical modelling techniques supporting diagnosis and treatment, and in screening programs of susceptible subpopulations, the financial investments for fertility treatment could be reduced.

Scientists all over the world demand for more action from governments, through funding and focussed research opportunities (18–20). There is a general agreement about a fundamental research gap in reproductive health. Compared to other clinical fields, research and funding opportunities to prevent or treat infertility has fallen behind. This may be due to other priorities, such as research on cancer, obesity-related disorders, and the emergence of COVID-19 in 2020. Although studies on these diseases are of high importance, the burden of infertility is well-established and expected to grow exponentially in the coming decades. Notably, subfertility is associated with other health related issues, including metabolic diseases, cancer, and early mortality (21). Hence, evaluation of male fertility could serve as a prognostic biomarker of future disease risk, suggesting that a comprehensive approach would benefit multiple aspects of the patient's health. Furthermore, long-term health effects of ART in offspring are still a concern not completely elucidated (22). Studies comparing outcomes of ART use vs. spontaneous conception show differences in preterm birth rates and weight for gestational age on the short term, and a higher risk for cardiovascular diseases on the long-term in people born after ART (5). Other problems overlooked are psychiatric disorders. While ART tries to overcome age-related issues in couples, it has been shown through multiple studies that older fathers have a higher risk to father a child with a psychiatric disorder (23).

## Female age as a major prognostic factor in current ART prediction modelling

Because of a high financial burden and the observation that cumulative live-birth delivery rates decrease dramatically by female age (10), some countries impose age limits for females and a maximum number of cycles (24). While female age is generally accepted as a predominant prognostic factor, women of same age have different pregnancy outcomes. Several studies have tried to improve prediction modelling in ART and especially to ameliorate its outcome. As such, we also followed this female-only approach and searched for a wider set of prognostic maternal factors, including ovulation problems, gonadotrophin dose, transport problems, pituitary inhibition, and implantation problems. We performed a prognostic Random Forest modelling algorithm on 8,000 *in vitro* fertilization (IVF) cycles and concluded that this was still not sufficient to yield good prediction performance for pregnancy outcomes (25). More recent studies also including female data and several supervised machine learning algorithms provide promising perspectives (26–28). While this indicates that the field is eagerly searching for an optimal tool to predict live birth before ART, the list of predictors remains relatively short and incomplete. Male characteristics and lifestyle factors of both partners are not considered in these novel mathematical models. Preconceptional data is generally limited to the age of the female partner. Next, these studies remain rather exploratory, as they include data from only one centre or geographical area. Finally, current clinical studies do not consider genetic inheritance of familial disorders or parental epigenetic profiles. In brief, these models cannot be used in decision making before the start of ART. They cannot adequately support clinicians in advising their patients whether it is worth starting treatment. We believe that prediction accuracy could be significantly increased if the number of selected features becomes higher -but well-thought- and based on scientific knowledge.

## A silent impact of men on a couple's pregnancy success

Increasing evidence support an underestimated effect from men on fertility success, in clinic and in the general population. Below we summarize factors with proven impact on pregnancy success. These determinants of sub- or infertility include intrinsic and extrinsic preconceptional exposures. If their roles on the sperm epigenome and semen quality are better understood prediction in clinic would become more accurate. Next, implementation of AI-based methodologies would help tackle the amount of information collected before or during treatment.

### The role of male age on infertility

While age of women is currently a major focus in ART decision-making, there is also a tendency that men delay fatherhood. In the US, paternal age at first child rose from 27.4 to 30.9 years, between 1972 and 2015 (29). It is known that advanced paternal age affects pregnancy success (30), and increases the risk for neuropsychiatric disorders in offspring; reviewed in *Pediatric Research* (23). Examples include a prospective study in 782 healthy couples (not attending the fertility clinic) where, regardless of the woman's age, men above the age of 30 had a significant reduction in the chance to father a child. More dramatic effects were seen in a subgroup of patients attending the fertility clinic (30). Our most recent research in an IVF setting suggests that male age interferes with embryo growth (31). Embryos grew slower with increasing age of the father, while no effect was seen by maternal age. Similar age-related effects have been shown in mice by other research groups. Older males produced fewer offspring and *in vitro* experiments showed adverse effects on blastocyst development and pregnancy success if males were older than 12 months (32). However, male age is currently not part of predictive models that are in regular use in ART.

### The effects of quality of sperm and embryo characteristics on pregnancy success

It is well known that the second most important factor affecting ART success is quality of oocyte, semen sample and embryo. However, also here most research is still female-oriented. Female age, egg productivity and embryo quality are major areas of attention in clinic. Besides IVF, incorporating techniques such as intracytoplasmic sperm injection (ICSI) are often used to by-pass issues of poor semen quality, but overall success rates remain disappointing (33, 34). Over the last decades multiple attempts have been performed to ameliorate predictions on ART outcomes through semen analyses before fertilization, but progress has been stagnated. Besides standard semen analyses, literature suggests to additionally assess sperm fragmentation assays after gradient centrifugation or swim-up. However, there is still no consensus about the optimal standard test method. For instance, the following methods are being used, but clearly defined guidelines and thresholds are missing: alkaline comet assay (detecting DNA strand breaks), terminal deoxynucleotidyl transferase-mediated dUTP-biotin nick end labelling (TUNEL) test (detecting DNA fragmentation), sperm chromatin structure assay (SCSA) or sperm chromatin dispersion test (SCD) (35). After six editions of a WHO-supported manual explaining how semen parameters of a normal man should be (36), experts agree that “*the current procedure of semen examination does not guarantee better prognosis, nor can it sufficiently predict success of embryo implantation and progress of a healthy full-term pregnancy”.* These were the concluding words at a recent International Webinar meeting of the European Academy of Andrology (EAA) (37).

Another important issue in ART is lack of evidence-based embryo grading methods. Embryo selection before implantation is generally based on a visual observation of morphologic characteristics by an embryologist. Although this method has been widely adopted for many years, recent studies have developed algorithms and computational models to improve this ranking system (38). However, these prediction models are often time-consuming and not always feasible, as they need expensive and elaborate imaging techniques (e.g., time-lapse imaging) (39). Most reports do not take into account variability of the patient population or preconceptional layers of exposures, sample sizes are limited (40), and validation tests on a large scale are missing (41). Numerous attempts have been performed to design the best-performing mathematical model to obtain successful pregnancy outcomes, and even the latest approaches including female allocation criteria to predict prognosis of “life birth”, are not satisfactory (42). More precisely, well-known Templeton and Nelson IVF-prediction models (43), and a latest adaptation of the Van Loendersloot prognostic model, do not answer all questions of patients and clinicians at different time points during treatment (44). In a review by Simopoulou et al., the authors conclude with the following discouraging note: “*It is unrealistic to expect that any of these models could successfully become a tool in the hands of embryologists or physicians*” (43). The field clearly needs a novel and scientifically supported approach. AI tools combined with a well-designed preprocessing of biological data may offer better prognostics (see our proposal below).

### The role of preconceptional obesity in future fathers on fertility

Potential risk factors from the preconceptional environment are currently not included in treatment trajectories. In some situations -such as if the mother is experiencing problems related to obesity- she is advised to improve her lifestyle before initiating ART. Although, this is not a general rule. Overall, patients in the fertility clinic are not better informed about influences from lifestyle factors on fertility, than the general population (45). This is certainly a missed opportunity in some clinics. Considering the effects of obesity, male infertility is most likely one of the underestimated “sequelae” of men living with obesity. For instance, through extensive systematic reviews, including a total of over 115,000 men, it has been shown that high BMI is associated with an increase in incidence of abnormal sperm count and a reduction in sperm motility (46, 47). Obese men are more likely to experience infertility (OR = 1.66, 95% CI: 1.53–1.79) and their rate of live birth per cycle of ART is significantly lower compared to men with a normal BMI (OR = 0.65, 95% CI: 0.44–0.97). Furthermore, obese men have a 10% absolute risk increase of pregnancy non-viability (47). An interesting observation is a co-occurring increase of obesity and infertility in certain regions of the world. For instance, prevalence of male obesity in Saudi Arabia increased from 18.5% in 1997 to 30.8% in 2016 (48). At the same time, the male factor contribution to infertility in the Middle East has become extremely high, 60%–70% (2). This is in line with our observation in a Flemish population of infertile men, recruited in a pilot IVF study between 2014 and 2017. We observed that 19.4% of men attending the fertility clinic was obese; while in the same period, only 8.5% of same age men was obese in the general population in Flanders (*p* < 0.001) (31). These results point to a possible influence of male obesity on a couple's fecundity. We believe this is even the case when semen parameters are clinically normal, as ICSI patients were excluded in our study.

Notably, clinical studies show that paternal weight not only lead to a decrease in fertility rate, but also it may also affect embryo kinetics (49). Other preconceptional conditions, such as tobacco smoking, alcohol or drug use, and occupational or environmental pollutants have also been suggested to harm embryo growth (50). These are factors not always questioned in clinic but discussed here below.

### Influences of smoking behaviour on male fertility

Evidence exists that smoking increases failure rates of ART. A German study in about 300 couples documented that odds ratios of IVF and ICSI failures for male smokers vs. non-smokers are 2.65 (95% CI: 1.33–5.30) and 2.95 (95% CI: 1.32–6.59), respectively (51). Results from a meta-analysis shows a significant association between cigarette smoking and reduced sperm quality, even if smoking behaviour was moderate (52). Next to conventional semen parameters, the sperm epigenome can also become affected by tobacco use. Jenkins et al. showed that DNA methylation patterns were different in sperm from smokers vs. non-smokers (53). This suggests that the germ cell epigenome is susceptible to toxins from cigarette smoking. As will be explained further, epigenetic “signatures” can persist through fertilization and influence embryo development and offspring health. In this context, two independent historical studies showed a link between paternal smoking and increased risk for obesity (54) and asthma in offspring (55). Other studies suggested that smoking of the father may also contribute to non-familial sporadic heritable retinoblastoma (56). However, more research is needed to confirm these results. Although long-term health outcomes in offspring are not the main scope of this review, potential disadvantages of paternal smoking before conception on next generation's health are noteworthy in the context of public health and prevention of diseases.

Interestingly, speculations based on biological recovery -and a “reset” of the sperm epigenome- such as from other harmful exposures to the ovary and germ cells (e.g., after chemotherapy) postulate that smoking cessation for a period of 3–6 months would be beneficial for a successful ART treatment (57). According to Vanegas et al., smoking cessation in future mothers -but also in future fathers- could reduce the probability of failing ART, particularly in favour of increased live birth rates (58). It has been suggested that in case men are unable to quit smoking, supplementation of antioxidants may be useful because smoking provokes a state of oxidative stress in the testes. However, these kinds of interventions have not sufficiently been tested yet and should be verified.

### A view on the effects of environmental chemicals and occupational exposures on male reproductive health

The general population is exposed to air pollution and environmental chemicals on a daily basis, such as endocrine disruptors (EDCs) found in personal care products (59), plastics or food packaging (60), and the surrounding environment (61). EDCs are known to disrupt the endocrine and metabolic homeostasis in the body, but other potential consequences include decreased reproductive function (60, 62, 63), neurodevelopmental delays in children (64), and increased risk of diabetes (65), or other chronic disorders via transgenerational inheritance of these exposures (66). A worrisome observation is the fact that exposure to EDCs has increased more than ten-fold over a period of ten years (61). Considering findings from animal experiments, showing that environmental chemicals can have short and long-term effects on reproductive health and next generations (67), the ubiquitous presence of chemicals may well tip the epigenetic balance and (re)program an individual for developing chronic conditions later in life, including infertility. Next to animal testing, there is a growing field of toxicology modelling, including the development of Adverse Outcome Pathways (AOPs). This framework provides a way to better understand biological responses and molecular effects from a specific chemical exposure. It is based on results from *in vitro* and *in silico* tests; but, the role of the epigenome has been understudied. Additionally, AOPs often misses information from longitudinal human studies and a potential link with infertility in human. We believe it is possible to integrate knowledge from these different fields -toxicology and AOP development, epigenetics and epidemiology- and create a novel comprehensive technology, which the fertility clinic urgently needs. For instance, before ART takes place, a short, structured survey or screening about earlier exposures could help to better understand the environment—sperm epigenome interaction and its impact on reproductive health. Especially in specific situations, such as through industrial pollution, specific lifestyle, nutritional conditions and/or a man's occupation. The latter may be an important unexplored reason for sub- or infertility, but still not in question when it comes to preconceptional care in couples. If these data are added in electronic health records, they could easily be added in prediction modelling when a couple seeks for treatment or undergo ART procedures. If not already implemented through a national or regional health care program, patients would benefit from an additional consultation provided by a counsellor skilled in biomedical sciences. In some cases, it may be advisable that men limit specific exposures at work or in their daily life (during a period of at least 3 months) before they conceive a child. Current policies at work are designed to protect future mothers and their child, but men remain out of scope.

## The sperm epigenome: a new diagnostic tool to better understand infertility and to predict pregnancy outcomes?

Despite the exponential growth in the field of machine learning, a recent call for more clinical studies testing predictive models demonstrates the need for improvement in multiple clinical areas (68). We believe that new insights in pre-conceptional male influences, and the role of sperm epigenetics in infertility would considerably help inform clinicians and patients in the fertility clinic. Changes in epigenetic patterns do not involve the genetic code, but include cellular mechanisms such as DNA methylation, histone modification, and expression of ncRNAs (non-coding RNAs). Changes in the epigenome play an important role in gene expression. For instance, dysregulation of DNA methylation (hypermethylation or hypomethylation) may contribute to infertility. The use of the sperm epigenome as a diagnostic tool in the fertility clinic has already been suggested (69, 70), but its implementation and especially the understanding of changing patterns in the epigenome over time -or in specific patients- is still in its infancy. We assume this is due to the many challenges this research involves. First, human infertility is a complex disorder and difficult to replicate in animal models (71). While experiments in laboratory animals have their limitations, studies in cattle support evidence that sperm transcriptome and methylome profiles may be useful in fertility assessment (72, 73). Second, in human studies, collecting exposure data in a prospective way is time consuming and a comprehensive approach is missing. The few studies exploring genome-wide epigenetic marks in human sperm to explore a link with infertility were based on cross-sectional designs and confounding factors were not considered (74). Moreover, case-control studies where infertile men were compared with fertile sperm donors did not match for age, or controls were selected within a specific clinical setting (75). Third, most infertility-related studies only focused on a small, predefined set of (imprinted) genes, such as *IGF2*, *H19*, *SNRPN* or *MEST* (76–78). Finally, to our knowledge only a few research groups assessed genome-wide methylation patterns in sperm related to male infertility, but results are not consistent (74, 75, 79, 80). In the meantime, commercial epigenetic tests in sperm have been brought on the market and may be promising (81). While this field is still evolving and novel epigenetic biomarkers related to infertility are being discovered (82), progress has also been made regarding the origin of epigenetic aberrancies in sperm cells and (potentially) associated male infertility. For instance, a limited number of independent genome-wide studies show that sperm DNA methylation is sensitive to ageing (83–88), obesity (89–91), smoking (53), the use of cannabis (92), and earlier neoadjuvant treatment (93). Men experience a wide range of (mixtures of) exposures throughout life, including age or lifestyle-related influences, intake of medication, exposure to occupational harmful substances and compounds of environmental pollutants (94, 95). We believe that identification of a well-defined set of epimutations as an intermediate link between specific exposures or “cocktails” of exposures and infertility, better knowledge about the attributed effects, and integration of these into mathematical modelling may facilitate prediction of pregnancy success. In this context, we earlier introduced a paradigm called “Paternal Origins of Health and Disease” or POHaD to stress the need for more research on the role of the father in (in)fertility and offspring health (90).

## Integration of epigenetic biomarkers, preconceptional and clinical information in novel mathematical modelling in the fertility clinic

Bringing different areas of research together, including epigenetics, epidemiology and innovative mathematical technologies would benefit the fertility clinic and its patients. Suggestions to apply epigenetic data in advanced machine learning analysis in similar medical fields sound promising (96). However, inclusion of epigenetic and preconceptional male features remains a rare and uncommon practice. If epigenetics is applied, areas of application include classification of diseases such as prostate cancer, metastatic brain tumours, breast cancer, neurodevelopmental syndromes, and coronary heart disease. Most studies used supervised machine learning methods, and the two most popular (and successful) methods tested were support vector machine (SVM) and random forest (97). Noticeably, a recent study tested a deep learning approach in several cancer types. Starting from expression patterns of 720 genes known to mediate epigenetic processes (“epifactors”) and a limited set of patient profiles, Cheng et al. used a Cox-nnet artificial neural network (ANN) framework to predicted patient outcomes, including a prognostic index (equivalent to hazard ratios) (98). Another recent effort to predict progression of Alzheimer's disease was designed by Chen et al. (99). Also here, a deep neural network-based model was able to outperform conventional machine learning approaches.

**Figure 1:**
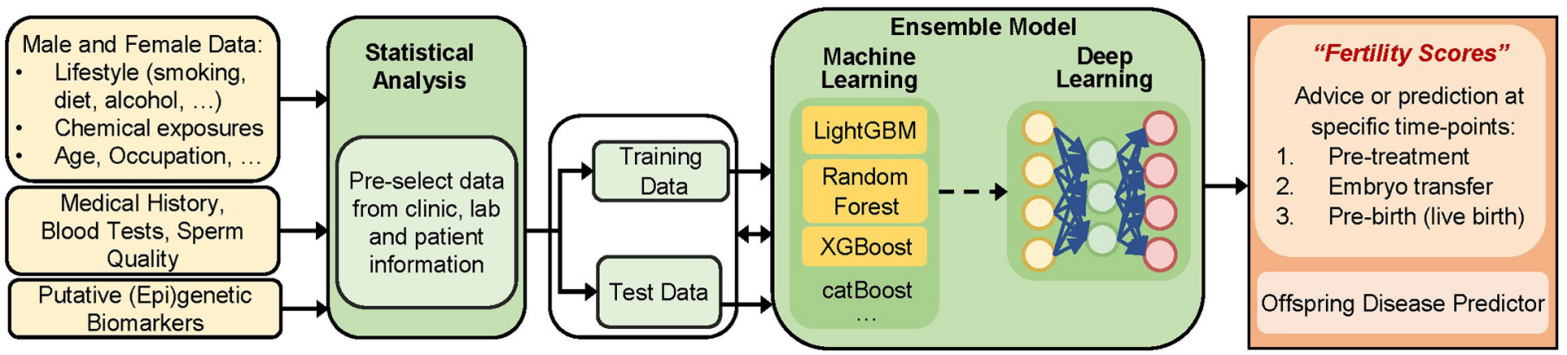
Building of a mathematical model to predict outcomes in a fertility setting, including data from clinic, laboratory test, sperm epigenetics, and preconceptional information from future mothers and fathers. The term “(epi)genetic biomarkers” can be interpreted as epigenetic modifications (such as DNA methylation levels or ncRNA expression profiles), but also as a set of genes mediating epigenetic processes. We propose using guided feature extraction steps (through scientific knowledge and human logic) before the creation of a predictive model. The way how this could be performed is via a stepwise approach using biostatistics as a pre-processing step (needed to carefully include biomarkers and/or select data based on specified criteria, cutoffs, magnitudes of change, etc.), followed by AI-based technologies and external validation testing (not shown). This will finally provide output at different levels: supporting patients, clinicians and embryologists at different time points, before treatment (providing an answer about the best treatment option), during treatment (embryo selection), after implantation (pregnancy success), and finally (if relevant) at birth (offspring health).

In the area of reproductive health, Tournaye et al. proposed in 2017 the creation of novel diagnostic algorithms with a stronger emphasis on genetics. However to our knowledge, their concept has not been further developed thus far (100). We believe that a combined approach would be highly effective. For instance, inclusion of a theoretical model—a traditional regression-based biostatistical model or the use of multiscale models- in feature selection for ML modelling would leverage current treatment strategies. This synergism may also offer a better prediction performance and decrease the computational power needed (101, 102). We here propose to fill the gap by carefully assessing and evaluating novel models in the fertility clinic (illustrated in Figure 1). Such models need a specific approach considering challenges and needs inherent to ART and its patients. For instance, ART records may include some complexity; hence, currently used off-the-shelf machine learning (ML) methods will not suffice to answer questions in this couple-oriented setting. To provide a personalized prognosis (e.g., taking into account male and female preconceptional data), new methods are needed to handle complicated datasets with various data types, including categorical, ordinal, numerical, time-to-event, missing data, etc., and to approach clustering of the data (e.g., multiple embryos per patient, parity, multiple cycles at different time points, fresh or thawed cycles, etc.). Next, regardless of the format and complexity of the available large data sets in clinic, incorporation of knowledge on biological processes or interactions is mostly based on literature, animal experiments and often few human data (e.g., from small sample sizes, but with high-dimensional information). A current challenge is selection of the most relevant variables needed to predict the outcome of interest accurately, especially when including epigenetic datasets. We suggest performing pre-processing procedures through classical statistical analysis on experimentally available data and/or publicly available data on methylome, transcriptome, or proteome (depending on the outcome of interest). After identification of a set of specific biomarkers, integration of such epigenetic markers in ML analysis would be highly informative to advise patients at specific important timepoints. First, before the start of treatment (or at any point during treatment) these theory-driven ML models could help to decide on the best treatment strategy. Second, these models could provide an individualized plan to improve a patient's chance of having a child, before or at the start of ART. Individual advice on potential reversibility of infertility is extremely valuable in this case. Third, the use of these models could help the embryologist in scoring or ranking embryos of a given couple. The use of complementary methodologies may also help identifying causality (101), which is an important goal in research on human infertility. For instance, state-of-the art combinations of computational models and algorithms could help define the attributed effects of earlier exposures or lifestyles on a person's ability to become a parent; hence, an individualized “fertility score” could be defined. Finally, we anticipate that further development in this field of research will provide valuable information to the general public about male factors that are related to failed pregnancies. Another interesting future application one can envision—once epigenetic biomarkers of disease inheritance are being developed—is prediction of offspring health and disease.

Obviously, external validation tests will be needed before implantation in clinic. To obtain a tool that supports healthcare professionals and improve patient care, the following processes are needed: (1) models need to operate on a hospital record system and extract relevant patient information; (2) models need to be integrated in a clinical workstation used by the treating physician (nurse or counsellor) and result in a user-friendly interface. For instance, feature selection procedures may help generate novel models that can operate with more sparse data sets.

## Conclusion

While ART has been the ultimate solution to by-pass infertility for over forty years, in only half of the cases treatment is successful. The fertility clinic is facing a major problem. We believe this is due to several reason: (1) there is a female-only approach in most preconceptional clinical practices; (2) the field is missing evidence-based prognostic factors based on male and female characteristics; (3) there is limited understanding of the etiology of infertility in most (male) patients and clinics are generally treatment-oriented; (4) there is still limited translation from recent findings in animal models to the fertility clinic; (5) clinicians have insufficient insights on preconceptional male influences and underlying epigenetic mechanisms; (6) new insights in prediction modelling are growing, although this area of study is still in its infancy, and the field is equally missing a golden standard in embryo selection or modelling. As suggested in the current review, these major challenges in clinic could be circumvented by implementation of novel epigenetic insights in environment-fertility relationships, male and female characteristics, and using state-of-the-art combinations of computational mathematics and modelling. Via this transdisciplinary approach, it would be possible to develop a model that estimates the chance to successfully conceive at different time points, such as before the start of treatment and at every step of treatment. It would provide an optimal individual advice on potential reversibility of infertility, informed by a multidisciplinary team; and, it would help to increase success of embryo selection once treatment has started.

Next to an improved and adequate patient care, further research and novel insights bridging traditional statistical modelling with the generation of ML algorithms would equally inform policy makers how to tailor an evidence-based prevention program. Australia is one of the few countries who have built a framework to work towards a “National men's health strategy” plan (103). They mainly focus on health promotion programs for prospective young fathers. As indicated above, infertility is a complex disorder. It could be a consequence of harmful exposures earlier in life, but it can also reflect other conditions not yet clinically relevant. Hence, better insights and improvement in modelling may have important implications, in clinic and in the context of preventative healthcare measures.

## Data Availability

The original contributions presented in the study are included in the article/Supplementary Material, further inquiries can be directed to the corresponding author.

## References

[1] WHO. Infertility Facts. (2021). Available online at: https://www.who.int/health-topics/infertility#tab=tab_2 (Cited December 9, 2021).

[2] Agarwal A Mulgund A Hamada A Chyatte MR. A unique view on male infertility around the globe. Reprod Biol Endocrinol. (2015) 13:37. 10.1186/s12958-015-0032-1 25928197 PMC4424520

[3] Babakhanzadeh E Nazari M Ghasemifar S Khodadadian A. Some of the factors involved in male infertility: a prospective review. Int J Gen Med. (2020) 13:29–41. 10.2147/IJGM.S241099 32104049 PMC7008178

[4] Steptoe PC Edwards RG. Birth after the reimplantation of a human embryo. Lancet. (1978) 312(8085):366. 10.1016/S0140-6736(78)92957-4 79723

[5] Pinborg A Wennerholm UB Bergh C. Long-term outcomes for children conceived by assisted reproductive technology. Fertil Steril. (2023) 120(3 Pt 1):449–56. 10.1016/j.fertnstert.2023.04.022 37086833

[6] Fauser BCJM Adamson GD Boivin J Chambers GM de Geyter C Dyer S Declining global fertility rates and the implications for family planning and family building: an IFFS consensus document based on a narrative review of the literature. Hum Reprod Update. (2024) 30(2):153–73. 10.1093/humupd/dmad028 38197291 PMC10905510

[7] Brandes M van der Steen JOM Bokdam SB Hamilton CJCM de Bruin JP Nelen WLDM When and why do subfertile couples discontinue their fertility care? A longitudinal cohort study in a secondary care subfertility population. Hum Reprod. (2009) 24(12):3127–35. 10.1093/humrep/dep340 19783833

[8] Engmann L Maconochie N Bekir JS Jacobs HS Tan SL. Cumulative probability of clinical pregnancy and live birth after a multiple cycle IVF package: a more realistic assessment of overall and age-specific success rates? Br J Obstet Gynaecol. (1999) 106(2):165–70. 10.1111/j.1471-0528.1999.tb08217.x 10426683

[9] Elizur SE Lerner-Geva L Levron J Shulman A Bider D Dor J. Cumulative live birth rate following *in vitro* fertilization: study of 5,310 cycles. Gynecol Endocrinol. (2006) 22(1):25–30. 10.1080/09513590500453916 16522530

[10] De Neubourg D Daels C Elseviers M Mangelschots K Vercruyssen M Van Royen E. Cumulative live-birth delivery after IVF/ICSI since the progressive introduction of single-embryo transfer. Reprod Biomed Online. (2010) 20(6):836–42. 10.1016/j.rbmo.2010.02.013 20362511

[11] De Neubourg D Bogaerts K Blockeel C Coetsier T Delvigne A Devreker F How do cumulative live birth rates and cumulative multiple live birth rates over complete courses of assisted reproductive technology treatment per woman compare among registries? Hum Reprod. (2016) 31(1):93–9. 10.1093/humrep/dev270 26537922

[12] Troude P Santin G Guibert J Bouyer J de La Rochebrochard E. Seven out of 10 couples treated by IVF achieve parenthood following either treatment, natural conception or adoption. Reprod Biomed Online. (2016) 33(5):560–7. 10.1016/j.rbmo.2016.08.010 27616620

[13] Bodri D Kawachiya S Brucker MD Tournaye H Kondo M Kato R Cumulative success rates following mild IVF in unselected infertile patients: a 3-year, single-centre cohort study. Reprod Biomed Online. (2014) 28(5):572–81. 10.1016/j.rbmo.2014.01.002 24631167

[14] Matorras R Chaudhari VS Roeder C Schwarze JE Bühler K Hwang K Evaluation of costs associated with fertility treatment leading to a live birth after one fresh transfer: a global perspective. Best Pract Res Clin Obstet Gynaecol. (2023) 89:102349. 10.1016/j.bpobgyn.2023.102349 37327667

[15] Chambers GM Sullivan EA Ishihara O Chapman MG Adamson GD. The economic impact of assisted reproductive technology: a review of selected developed countries. Fertil Steril. (2009) 91(6):2281–94. 10.1016/j.fertnstert.2009.04.029 19481642

[16] Lemos EV Zhang D Van Voorhis BJ Hu XH. Healthcare expenses associated with multiple vs singleton pregnancies in the United States. Am J Obstet Gynecol. (2013) 209(6):586.e1–e11. 10.1016/j.ajog.2013.10.005 24238479

[17] Crawford S Boulet SL Mneimneh AS Perkins KM Jamieson DJ Zhang Y Costs of achieving live birth from assisted reproductive technology: a comparison of sequential single and double embryo transfer approaches. Fertil Steril. (2016) 105(2):444–50. 10.1016/j.fertnstert.2015.10.032 26604068 PMC5125029

[18] Leung AK Henry MA Mehta A. Gaps in male infertility health services research. Transl Androl Urol. (2018) 7(Suppl 3):S303–9. 10.21037/tau.2018.05.03 30159236 PMC6087843

[19] Al-Kandari AM Alenezi A. Cost burden of male infertility investigations and treatments: a survey study. Urol Ann. (2020) 12(4):314–8. 10.4103/UA.UA_48_20 33776325 PMC7992531

[20] Mercuri ND Cox BJ. The need for more research into reproductive health and disease. Elife. (2022) 11:e75061. 10.7554/eLife.75061 36511240 PMC9771341

[21] Chen T Belladelli F Giudice FD Eisenberg ML. Male fertility as a marker for health. Reprod Biomed Online. (2021) 44(1):131–44. 10.1016/j.rbmo.2021.09.023 34848151

[22] Ahmadi H Aghebati-Maleki L Rashidiani S Csabai T Nnaemeka OB Szekeres-Bartho J. Long-term effects of ART on the health of the offspring. Int J Mol Sci. (2023) 24(17):13564. 10.3390/ijms241713564 37686370 PMC10487905

[23] Vervoort I Delger C Soubry A. A multifactorial model for the etiology of neuropsychiatric disorders: the role of advanced paternal age. Pediatr Res. (2022) 91(4):757–70. 10.1038/s41390-021-01435-4 33674740

[24] KB. Koninklijk Besluit Houdende Invoering van een Forfaitaire Tegemoetkoming Voor de Behandeling van Vruchtbaarheidsstoornissen bij Vrouwen. Belgium: Royal State Book (2008).

[25] Wang S Molenberghs G Spiessens C De Neubourg D Soubry A. Predicting pregnancy rate and live birth rate in the IVF clinic by analyzing patient profiles. Arch Public Health. (2015) 73:46. 10.1186/2049-3258-73-S1-P4626623009

[26] Qiu J Li P Dong M Xin X Tan J. Personalized prediction of live birth prior to the first *in vitro* fertilization treatment: a machine learning method. J Transl Med. (2019) 17(1):317. 10.1186/s12967-019-2062-5 31547822 PMC6757430

[27] Goyal A Kuchana M Ayyagari KPR. Machine learning predicts live-birth occurrence before *in vitro* fertilization treatment. Sci Rep. (2020) 10(1):20925. 10.1038/s41598-020-76928-z 33262383 PMC7708502

[28] McLernon DJ Steyerberg EW te Velde ER Lee AJ Bhattacharya S. Predicting the chances of a live birth after one or more complete cycles of *in vitro* fertilisation: population based study of linked cycle data from 113 873 women. Br Med J. (2016) 355:i5735. 10.1136/bmj.i5735 27852632 PMC5112178

[29] Khandwala YS Zhang CA Lu Y Eisenberg ML. The age of fathers in the USA is rising: an analysis of 168 867 480 births from 1972 to 2015. Hum Reprod. (2017) 32(10):2110–6. 10.1093/humrep/dex267 28938735

[30] de La Rochebrochard E de Mouzon J Thépot F Thonneau P. Fathers over 40 and increased failure to conceive: the lessons of *in vitro* fertilization in France. Fertil Steril. (2006) 85(5):1420–4. 10.1016/j.fertnstert.2005.11.040 16616749

[31] Van Opstal J Fieuws S Spiessens C Soubry A. Male age interferes with embryo growth in IVF treatment. Hum Reprod. (2021) 36(1):107–15. 10.1093/humrep/deaa256 33164068

[32] Katz-Jaffe MG Parks J McCallie B Schoolcraft WB. Aging sperm negatively impacts *in vivo* and *in vitro* reproduction: a longitudinal murine study. Fertil Steril. (2013) 100(1):262–8.e2. 10.1016/j.fertnstert.2013.03.021 23579004

[33] BelRap. Assisted Reproductive Technology National Summary Report, Belgium (2015). [2017 June 2018]. Available online at: https://www.belrap.be/Documents/Reports/Global/BelrapSummaryReport2017_20191015.pdf

[34] CDC. ART Success Rates. (2019). Available online at: https://www.cdc.gov/art/artdata/index.html (January 28, 2022).

[35] Esteves SC Zini A Coward RM Evenson DP Gosálvez J Lewis SEM Sperm DNA fragmentation testing: summary evidence and clinical practice recommendations. Andrologia. (2021) 53(2):e13874. 10.1111/and.13874 33108829 PMC7988559

[36] WHO. WHO Laboratory Manual for the Examination and Processing of Human Semen. 6th ed. Geneva: World Health Organization (2021).

[37] EAA. 2022 (January 18, 2022).

[38] Bormann CL Thirumalaraju P Kanakasabapathy MK Kandula H Souter I Dimitriadis I Consistency and objectivity of automated embryo assessments using deep neural networks. Fertil Steril. (2020) 113(4):781–787.e1. 10.1016/j.fertnstert.2019.12.004 32228880 PMC7583085

[39] Huang Bo Zheng S Ma B Yang Y Zhang S Jin L. Using deep learning to predict the outcome of live birth from more than 10,000 embryo data. BMC Pregnancy Childbirth. (2022) 22(1):36. 10.1186/s12884-021-04373-5 35034623 PMC8761300

[40] Vogiatzi P Pouliakis A Siristatidis C. An artificial neural network for the prediction of assisted reproduction outcome. J Assist Reprod Genet. (2019) 36(7):1441–8. 10.1007/s10815-019-01498-7 31218565 PMC6642243

[41] Loewke K Cho JH Brumar CD Maeder-York P Barash O Malmsten JE Characterization of an artificial intelligence model for ranking static images of blastocyst stage embryos. Fertil Steril. (2022) 117(3):528–35. 10.1016/j.fertnstert.2021.11.022 34998577

[42] Li F Lu R Zeng C Li X Xue Q. Development and validation of a clinical pregnancy failure prediction model for poor ovarian responders during IVF/ICSI. Front Endocrinol. (2021) 12:717288. 10.3389/fendo.2021.717288 PMC8419272 34497586

[43] Simopoulou M Sfakianoudis K Antoniou N Maziotis E Rapani A Bakas P Making IVF more effective through the evolution of prediction models: is prognosis the missing piece of the puzzle? Syst Biol Reprod Med. (2018) 64(5):305–23. 10.1080/19396368.2018.1504347 30088950

[44] Devroe J Peeraer K Verbeke G Spiessens C Vriens J Dancet E. Predicting the chance on live birth per cycle at each step of the IVF journey: external validation and update of the van Loendersloot multivariable prognostic model. BMJ Open. (2020) 10(10):e037289. 10.1136/bmjopen-2020-037289 33033089 PMC7545639

[45] van Asseldonk F. *Fertility patients' awareness on the effect of lifestyle and maternal age on pregnancy and miscarriage rates* (thesis in public health). Leuven, Belgium: KULeuven University (2013). 51p.

[46] Sermondade N Faure C Fezeu L Shayeb AG Bonde JP Jensen TK BMI In relation to sperm count: an updated systematic review and collaborative meta-analysis. Hum Reprod Update. (2013) 19(3):221–31. 10.1093/humupd/dms050 23242914 PMC3621293

[47] Campbell JM Lane M Owens JA Bakos HW. Paternal obesity negatively affects male fertility and assisted reproduction outcomes: a systematic review and meta-analysis. Reprod Biomed Online. (2015) 31(5):593–604. 10.1016/j.rbmo.2015.07.012 26380863

[48] Althumiri NA Basyouni MH AlMousa N AlJuwaysim MF Almubark RA BinDhim NF Obesity in Saudi Arabia in 2020: prevalence, distribution, and its current association with Various health conditions. Healthcare. (2021) 9(3):311. 10.3390/healthcare9030311 33799725 PMC7999834

[49] Bellver J. Obesity and poor reproductive outcome: female and male body weight matter. Fertil Steril. (2013) 99(6):1558–9. 10.1016/j.fertnstert.2013.01.142 23461824

[50] Barratt CLR Björndahl L De Jonge CJ Lamb DJ Osorio Martini F McLachlan R The diagnosis of male infertility: an analysis of the evidence to support the development of global WHO guidance-challenges and future research opportunities. Hum Reprod Update. (2017) 23(6):660–80. 10.1093/humupd/dmx021 28981651 PMC5850791

[51] Zitzmann M Rolf C Nordhoff V Schräder G Rickert-Föhring M Gassner P Male smokers have a decreased success rate for *in vitro* fertilization and intracytoplasmic sperm injection. Fertil Steril. (2003) 79(Suppl 3):1550–4. 10.1016/S0015-0282(03)00339-X 12801558

[52] Sharma R Harlev A Agarwal A Esteves SC. Cigarette smoking and semen quality: a new meta-analysis examining the effect of the 2010 world health organization laboratory methods for the examination of human semen. Eur Urol. (2016) 70(4):635–45. 10.1016/j.eururo.2016.04.010 27113031

[53] Jenkins TG James ER Alonso DF Hoidal JR Murphy PJ Hotaling JM Cigarette smoking significantly alters sperm DNA methylation patterns. Andrology. (2017) 5(6):1089–99. 10.1111/andr.12416 28950428 PMC5679018

[54] Northstone K Golding J Davey Smith G Miller LL Pembrey M. Prepubertal start of father’s Smoking and increased body fat in his sons: further characterisation of paternal transgenerational responses. Eur J Hum Genet. (2014) 22(12):1382–6. 10.1038/ejhg.2014.31 24690679 PMC4085023

[55] Accordini S Calciano L Johannessen A Portas L Benediktsdottir B Bertelsen RJ A three-generation study on the association of tobacco smoking with asthma. Int J Epidemiol. (2018) 47(4):1106–17. 10.1093/ije/dyy031 29534228 PMC6124624

[56] Kumar SB Chawla B Bisht S Yadav RK Dada R. Tobacco use increases oxidative DNA damage in sperm—possible etiology of childhood cancer. Asian Pac J Cancer Prev. (2015) 16(16):6967–72. 10.7314/APJCP.2015.16.16.6967 26514476

[57] de Ziegler D Santulli P Seroka A Decanter C Meldrum DR Chapron C. In women, the reproductive harm of toxins such as tobacco smoke is reversible in 6 months: basis for the “olive tree” hypothesis. Fertil Steril. (2013) 100(4):927–8. 10.1016/j.fertnstert.2013.05.043 23796366

[58] Vanegas JC Chavarro JE Williams PL Ford JB Toth TL Hauser R Discrete survival model analysis of a couple’s smoking pattern and outcomes of assisted reproduction. Fertil Res Pract. (2017) 3:5. 10.1186/s40738-017-0032-2 28480049 PMC5416813

[59] Philippat C Bennett D Calafat AM Picciotto IH. Exposure to select phthalates and phenols through use of personal care products among Californian adults and their children. Environ Res. (2015) 140:369–76. 10.1016/j.envres.2015.04.009 25929801 PMC4724203

[60] Lee E Ahn MY Kim HJ Kim IY Han SY Kang TS Effect of di(n-butyl) phthalate on testicular oxidative damage and antioxidant enzymes in hyperthyroid rats. Environ Toxicol. (2007) 22(3):245–55. 10.1002/tox.20259 17497641

[61] Hoffman K Butt CM Webster TF Preston EV Hammel SC Makey C Temporal trends in exposure to organophosphate flame retardants in the United States. Environ Sci Technol Lett. (2017) 4(3):112–8. 10.1021/acs.estlett.6b00475 28317001 PMC5352975

[62] Anway MD Cupp AS Uzumcu M Skinner MK. Epigenetic transgenerational actions of endocrine disruptors and male fertility. Science. (2005) 308(5727):1466–9. 10.1126/science.1108190 15933200 PMC11423801

[63] Buck Louis GM Sundaram R Sweeney AM Schisterman EF Maisog J Kannan K. Urinary bisphenol A, phthalates, and couple fecundity: the longitudinal investigation of fertility and the environment (LIFE) study. Fertil Steril. (2014) 101(5):1359–66. 10.1016/j.fertnstert.2014.01.022 24534276 PMC4008721

[64] Braun JM. Early-life exposure to EDCs: role in childhood obesity and neurodevelopment. Nat Rev Endocrinol. (2017) 13(3):161–73. 10.1038/nrendo.2016.186 27857130 PMC5322271

[65] Bonini MG Sargis RM. Environmental toxicant exposures and type 2 diabetes Mellitus: two interrelated public health problems on the rise. Curr Opin Toxicol. (2018) 7:52–9. 10.1016/j.cotox.2017.09.003 29392186 PMC5788318

[66] Guerrero-Bosagna C Covert TR Haque MM Settles M Nilsson EE Anway MD Epigenetic transgenerational inheritance of vinclozolin induced mouse adult onset disease and associated sperm epigenome biomarkers. Reprod Toxicol. (2012) 34(4):694–707. 10.1016/j.reprotox.2012.09.005 23041264 PMC3513496

[67] Skinner MK Manikkam M Guerrero-Bosagna C. Epigenetic transgenerational actions of endocrine disruptors. Reprod Toxicol. (2011) 31(3):337–43. 10.1016/j.reprotox.2010.10.012 21055462 PMC3068236

[68] Fihn SD Berlin JA Haneuse SJPA Rivara FP. Prediction models and clinical outcomes-A call for papers. JAMA Netw Open. (2024) 7(4):e249640. 10.1001/jamanetworkopen.2024.9640 38607631

[69] Filipponi D Feil R. Perturbation of genomic imprinting in oligozoospermia. Epigenetics. (2009) 4(1):27–30. 10.4161/epi.4.1.7311 19106644

[70] Jenkins TG Aston KI James ER Carrell DT. Sperm epigenetics in the study of male fertility, offspring health, and potential clinical applications. Syst Biol Reprod Med. (2017) 63(2):69–76. 10.1080/19396368.2016.1274791 28301256

[71] Jamsai D O'Bryan MK. Mouse models in male fertility research. Asian J Androl. (2011) 13(1):139–51. 10.1038/aja.2010.101 21057516 PMC3739380

[72] Lalancette C Thibault C Bachand I Caron N Bissonnette N. Transcriptome analysis of bull semen with extreme nonreturn rate: use of suppression-subtractive hybridization to identify functional markers for fertility. Biol Reprod. (2008) 78(4):618–35. 10.1095/biolreprod.106.059030 18003951

[73] Costes V Chaulot-Talmon A Sellem E Perrier J-P Aubert-Frambourg A Jouneau L Predicting male fertility from the sperm methylome: application to 120 bulls with hundreds of artificial insemination records. Clin Epigenetics. (2022) 14(1):54. 10.1186/s13148-022-01275-x 35477426 PMC9047354

[74] Laqqan M Hammadeh ME. Aberrations in sperm DNA methylation patterns of males suffering from reduced fecundity. Andrologia. (2018) 50(3):e12913. 10.1111/and.12913 29072328

[75] Jenkins TG Aston KI Meyer TD Hotaling JM Shamsi MB Johnstone EB Decreased fecundity and sperm DNA methylation patterns. Fertil Steril. (2016) 105(1):51–7.e3. 10.1016/j.fertnstert.2015.09.013 26453269 PMC4890464

[76] Song B Wang C Chen Y Li G Gao Y Zhu F Sperm DNA integrity status is associated with DNA methylation signatures of imprinted genes and non-imprinted genes. J Assist Reprod Genet. (2021) 38(8):2041–8. 10.1007/s10815-021-02157-6 33786731 PMC8417181

[77] Marques CJ Francisco T Sousa S Carvalho F Barros A Sousa M. Methylation defects of imprinted genes in human testicular spermatozoa. Fertil Steril. (2010) 94(2):585–94. 10.1016/j.fertnstert.2009.02.051 19338988

[78] Poplinski A Tüttelmann F Kanber D Horsthemke B Gromoll J. Idiopathic male infertility is strongly associated with aberrant methylation of MEST and IGF2/H19 ICR1. Int J Androl. (2010) 33(4):642–9. 10.1111/j.1365-2605.2009.01000.x 19878521

[79] Aston KI Uren PJ Jenkins TG Horsager A Cairns BR Smith AD Aberrant sperm DNA methylation predicts male fertility status and embryo quality. Fertil Steril. (2015) 104(6):1388–1397.e5. 10.1016/j.fertnstert.2015.08.019 26361204

[80] Leitão E Di Persio S Laurentino S Wöste M Dugas M Kliesch S The sperm epigenome does not display recurrent epimutations in patients with severely impaired spermatogenesis. Clin Epigenetics. (2020) 12(1):61. 10.1186/s13148-020-00854-0 32375885 PMC7204326

[81] Brogaard K Schneider L Olson A. Economic benefit of an epigenetic male infertility assessment. ASRM 2024 Scientific Congress (2024). p. 122

[82] Miller RH DeVilbiss EA Brogaard KR Norton CR Pollard CA Emery BR Epigenetic determinants of reproductive potential augment the predictive ability of the semen analysis. F S Sci. (2023) 4(4):279–85. 10.1016/j.xfss.2023.09.001 37714409 PMC10843460

[83] Oluwayiose OA Wu H Saddiki H Whitcomb BW Balzer LB Brandon N Sperm DNA methylation mediates the association of male age on reproductive outcomes among couples undergoing infertility treatment. Sci Rep. (2021) 11(1):3216. 10.1038/s41598-020-80857-2 33547328 PMC7864951

[84] Jenkins TG Aston KI Cairns B Smith A Carrell DT. Paternal germ line aging: DNA methylation age prediction from human sperm. BMC Genomics. (2018) 19(1):763. 10.1186/s12864-018-5153-4 30348084 PMC6198359

[85] Bernhardt L Dittrich M Prell A Potabattula R Drummer C Behr R Age-related methylation changes in the human sperm epigenome. Aging (Albany NY). (2023) 15(5):1257–78. 10.18632/aging.204546 36849136 PMC10042684

[86] Denomme MM Haywood ME Parks JC Schoolcraft WB Katz-Jaffe MG. The inherited methylome landscape is directly altered with paternal aging and associated with offspring neurodevelopmental disorders. Aging Cell. (2020) 19:e13178. 10.1111/acel.13178 32610362 PMC7431824

[87] Laurentino S Cremers J-F Horsthemke B Tüttelmann F Czeloth K Zitzmann M A germ cell-specific ageing pattern in otherwise healthy men. Aging Cell. (2020) 19(10):e13242. 10.1111/acel.13242 32951333 PMC7576283

[88] Lee HY Jung S-E Oh YN Choi A Yang WI Shin K-J. Epigenetic age signatures in the forensically relevant body fluid of semen: a preliminary study. Forensic Sci Int Genet. (2015) 19:28–34. 10.1016/j.fsigen.2015.05.014 26057119

[89] Keyhan S Burke E Schrott R Huang Z Grenier C Price T Male obesity impacts DNA methylation reprogramming in sperm. Clin Epigenetics. (2021) 13(1):17. 10.1186/s13148-020-00997-0 33494820 PMC7831195

[90] Donkin I Versteyhe S Ingerslev LR Qian K Mechta M Nordkap L Obesity and bariatric surgery drive epigenetic variation of spermatozoa in humans. Cell Metab. (2016) 23(2):369–78. 10.1016/j.cmet.2015.11.004 26669700

[91] Soubry A Guo L Huang Z Hoyo C Romanus S Price T Obesity-related DNA methylation at imprinted genes in human sperm: results from the TIEGER study. Clin Epigenetics. (2016) 8:51. 10.1186/s13148-016-0217-2 27158277 PMC4859994

[92] Schrott R Murphy SK Modliszewski JL King DE Hill B Itchon-Ramos N Refraining from use diminishes cannabis-associated epigenetic changes in human sperm. Environ Epigenet. (2021) 7(1):dvab009. 10.1093/eep/dvab009 34557312 PMC8455898

[93] Shnorhavorian M Schwartz SM Stansfeld B Sadler-Riggleman I Beck D Skinner MK. Differential DNA methylation regions in adult human sperm following adolescent chemotherapy: potential for epigenetic inheritance. PLoS One. (2017) 12(2):e0170085. 10.1371/journal.pone.0170085 28146567 PMC5287489

[94] Soubry A Hoyo C Butt CM Fieuws S Price TM Murphy SK Human exposure to flame-retardants is associated with aberrant DNA methylation at imprinted genes in sperm. Environ Epigenet. (2017) 3(1):dvx003. 10.1093/eep/dvx003 29492305 PMC5804543

[95] Soubry A. POHad: why we should study future fathers. Environ Epigenet. (2018) 4(2):dvy007. 10.1093/eep/dvy007 29732171 PMC5920283

[96] Holder LB Haque MM Skinner MK. Machine learning for epigenetics and future medical applications. Epigenetics. (2017) 12(7):505–14. 10.1080/15592294.2017.1329068 28524769 PMC5687335

[97] Rauschert S Raubenheimer K Melton PE Huang RC. Machine learning and clinical epigenetics: a review of challenges for diagnosis and classification. Clin Epigenetics. (2020) 12(1):51. 10.1186/s13148-020-00842-4 32245523 PMC7118917

[98] Cheng MW Mitra M Coller HA. Pan-cancer landscape of epigenetic factor expression predicts tumor outcome. Commun Biol. (2023) 6(1):1138. 10.1038/s42003-023-05459-w 37973839 PMC10654613

[99] Chen Li Saykin AJ Yao B Zhao F. Multi-task deep autoencoder to predict Alzheimer’s disease progression using temporal DNA methylation data in peripheral blood. Comput Struct Biotechnol J. (2022) 20:5761–74. 10.1016/j.csbj.2022.10.016 36756173 PMC9619306

[100] Tournaye H Krausz C Oates RD. Novel concepts in the aetiology of male reproductive impairment. Lancet Diabetes Endocrinol. (2017) 5(7):544–53. 10.1016/S2213-8587(16)30040-7 27395771

[101] Alber M Buganza Tepole A Cannon WR De S Dura-Bernal S Garikipati K Integrating machine learning and multiscale modeling-perspectives, challenges, and opportunities in the biological, biomedical, and behavioral sciences. NPJ Digit Med. (2019) 2:115. 10.1038/s41746-019-0193-y 31799423 PMC6877584

[102] Eisbach A Mai O Hertel G. Combining theoretical modelling and machine learning approaches: the case of teamwork effects on individual effort expenditure. New Ideas Psychol. (2024) 73:101077. 10.1016/j.newideapsych.2024.101077

[103] Australia, C.o. In: D.o. Health, editor. National Men’s Health Strategy 2020–2030. Canberra, Australia: Health Government Australia (2019). p. 1–42.

